# Role of D-mannose in urinary tract infections – a narrative review

**DOI:** 10.1186/s12937-022-00769-x

**Published:** 2022-03-22

**Authors:** Reeta Ala-Jaakkola, Arja Laitila, Arthur C. Ouwehand, Liisa Lehtoranta

**Affiliations:** Health & Biosciences, International Flavors & Fragrances, Sokeritehtaantie 20, FIN-02460 Kantvik, Finland

**Keywords:** D-mannose, UTI, Urinary tract infection, *Escherichia coli*

## Abstract

Urinary tract infections (UTIs) are one of the most prevalent bacterial diseases worldwide. Despite the efficacy of antibiotics targeted against UTI, the recurrence rates remain significant among the patients. Furthermore, the development of antibiotic resistance is a major concern and creates a demand for alternative treatment options. D-mannose, a monosaccharide naturally found in fruits, is commonly marketed as a dietary supplement for reducing the risk for UTIs. Research suggests that supplemented D-mannose could be a promising alternative or complementary remedy especially as a prophylaxis for recurrent UTIs. When excreted in urine, D-mannose potentially inhibits *Escherichia coli*, the main causative organism of UTIs, from attaching to urothelium and causing infection. In this review, we provide an overview of UTIs, *E. coli* pathogenesis and D-mannose and outline the existing clinical evidence of D-mannose in reducing the risk of UTI and its recurrence. Furthermore, we discuss the potential effect mechanisms of D-mannose against uropathogenic *E.coli*.

## Introduction

Urinary tract infections (UTIs) are among the leading infectious diseases globally. UTIs are highly prevalent in women, especially after menopause. Despite the short-term impact of antibiotics on acute UTIs, a long-term risk of recurrence still exists. Furthermore, antibiotic resistance of UTI pathogens to many commonly used antimicrobial drugs is alarmingly increasing. For instance, 90% of the UTI causing *Escherichia coli* strains in patients treated with trimethoprim-sulfamethoxazole for a month were resistant to the antibiotic, whereas in the control group, subject to cranberry juice, the incidence was 28% [1]. *E. coli* is the causative organism in 85% of UTI cases. The adhesion of *E. coli* in the urinary tract is mainly based on mannose-sensitive mechanism, where *E.coli* type I pili adhere to mannose structures on the uroepithelial cell surfaces [2, 3].

D-mannose is a monosaccharide, naturally found in various plants, and fruits/berries, for instance in cranberries. It is also known to be synthesized in the body from glucose for the synthesis of glycoproteins [4]. D-mannose is commonly marketed as a dietary supplement for urinary tract health. Research suggests that free D-mannose in urine has the potential to saturate *E. coli* FimH structures, and subsequently block *E. coli* adhesion to urinary tract epithelial cells. This so-called competitive inhibition is considered as one of the potential mechanisms for preventing UTI development [5].

The aim of this review was to examine the current evidence on the role of D-mannose against UTI. Earlier reviews have focused on various aspects of this topic. Here, we integrate these parts into one comprehensive narrative; presenting an overview of UTIs, urethral microbiota, current treatments and *E. coli* pathogenesis followed by D-mannose and its potential effect mechanisms against uropathogenic *E. coli* (UPEC). Finally, we review existing preclinical and clinical studies which have investigated D-mannose in UTIs.

## Overview of UTI

### Prevalence

A WHO report from 2017 listed *E. coli* as the main species responsible for community- and hospital- acquired UTIs [6]. The WHO has recognized the matter as a high community and health-care burden. More than 150 million people are affected by UTIs annually [7, 8]. It is considered as one of the most common infections in communities as well as within healthcare settings. The prevalence of UTIs are especially high among women. An estimated 11% of women over the age of 18 suffer from UTI annually [9]. Approximately 50% of all women will have at least one UTI episode during their lifetime [10]. Women are at risk for UTI due to a short urethra located close to the rectum, which allows easier access for bacteria to the urinary tract as compared to men. Changes in the sexual activity, pregnancy, and menopausal status have a high impact on the risk for UTI occurrence since all of them affect the urogenital bacterial composition. Higher prevalence to UTI is also seen among specific populations such as people with structural changes (e.g. prostate enlargement) and diabetics (up to 35% of the patients) [11–13]. Moreover, healthcare-associated UTIs are the most common infections occurring in intensive-care units, especially among patients needing catheterization [14]. Furthermore, UTI is listed among the 10 most common reasons for unplanned readmission to medical care [15]. Therefore, the societal and healthcare costs caused by hospitalizations and medical expenses associated with UTI are high.

### Diagnosis and etiology

UTIs can be categorized into several sub-classes based on their complexity, acuteness, and location [16]. Clinically, UTIs are classified as uncomplicated or complicated, where the first often considers otherwise healthy individuals and the latter is associated with structural or functional challenges e.g. pregnancy, male gender, young age (children), catheterization, or diabetes, which complicate the condition. UTI diagnosis can also be a recurrent UTI (rUTI) defined by the occurrence of more than 2 symptomatic UTIs within the last 6 months or more than 3 within the last 12 months. UTIs can be localized either in the upper urinary tract, including kidneys (upper UTI a.k.a. pyelonephritis), or on the lower urinary tract, affecting the bladder (lower UTI a.k.a. cystitis) [16].

The gold standard for UTI diagnosis is based on pathogen detection and identification from a midstream urine sample (10^3^–10^5^ or more colony forming units (CFU)/ml urine) combined with clinical symptoms (dysuria, frequency, urgency, suprapubic pain, nocturia, and hematuria). In case the clinical symptoms are absent, and the number of bacteria counts exceed 10^5^ CFU/ml, the diagnosis is asymptomatic bacteriuria and treatment is only rarely prescribed [17].

UPEC is the main causative organism of UTIs, in both uncomplicated and complicated infections, being the responsible pathogen in up to 85% of the cases. Other pathogenic microbes associated with uncomplicated UTIs are, starting from the most likely pathogen, *Klebsiella pneumoniae, Staphylococcus saprophyticus, Enterococcus faecalis*, Group B *Streptococcus* (GBS), *Proteus mirabilis, Pseudomonas aeruginosa, Staphylococcus aureus* as well as *Candida* species. Common pathogens associated with complicated UTIs are *Enterococcus* spp.*, K. pneumoniae, Candida* spp.*, S. aureus, P. mirabilis, P. aeruginosa*, and GBS [7].

### Urinary microbiota and UTI

Advancements in molecular techniques have increased the understanding of the microbial community in the urinary tract, which has been previously regarded as sterile [18]. Overall, in contrast to the gut, urine contains very few microbes and is dominated by one or two species (also called as urotypes) [18, 19]. Research implies that the urinary microbiota is gender specific, likely due to anatomical and hormonal differences [20, 21]. As women are more at risk of UTI, we mainly focus on providing an overview of the urinary microbiota of women and association with UTI.

The most common bacteria in the urinary microbiota of healthy women are the same species of *Lactobacillus* that exists in the vagina [18, 22]. Other predominating species are from the genera *Gardnerella*, *Streptococcus*, *Staphylococcus*, *Corynebacterium,* and *Escherichia*. Research suggests that urotype changes with age and for instance a *Lactobacillus-* or *Gardnerella-*dominated urotype is in some cases reported to be more common in pre-menopausal women, whereas the *Escherichia*-dominated urotype and more diverse microbiota seem to predominate in postmenopausal women [18, 23].

Urinary microbiota is associated with rUTIs [24]. Especially changes resulting in the loss of normally protective *Lactobacillus* spp. seem to increase the risk of UTI. The vaginal tract is suggested to play a role in UTI pathogenesis by serving as a potential reservoir for uropathogenic bacteria ascending from the gastrointestinal tract. Studies show that women with rUTI have lower abundance of lactobacilli and are more commonly colonized with vaginal *E. coli* [24, 25]. Indigenous vaginal lactobacilli produce H_2_O_2_ and lactic acid which contributes to lowering vaginal pH which thus inhibits the growth of pathogenic bacteria, such as *E. coli*, and may ultimately reduce the risk of such organisms colonizing the urinary tract.

### Pathogenesis

**Fig. 1:**
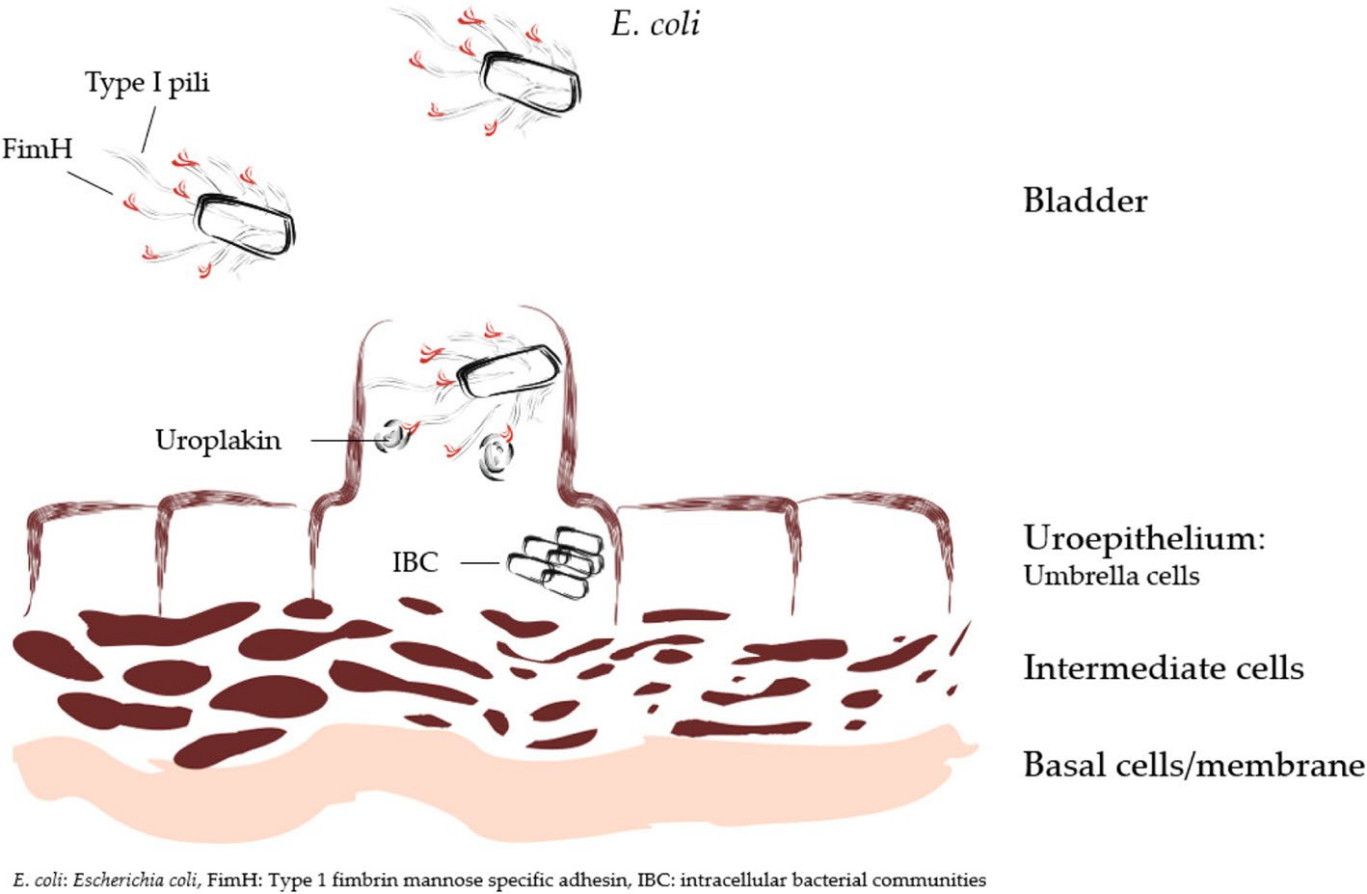
Schematic representation of *E. coli* attachment by FimH tips of the type 1 pili adhesins to mannosylated uroplakins on the surface of uroepithelium

The pathogenicity of UTI associated bacteria is based on their ability to attach, colonize, and survive in the urinary tract environment. UPEC strains, the most common pathogens for UTI, mainly enter the urogenital tract from the gut via fecal–perineal–urethral route [26]. UPEC strains possess several virulence factors, such as adhesins, toxins, iron acquisition factors, lipopolysacharide and capsules, that contribute to UTI pathogenesis. One of the main disease-causing mechanisms for UPEC is based on its adherence to mannosylated protein components called uroplakins on the bladder epithelium (Fig. 1) [2, 3]. This binding occurs via the FimH tip of the type I pili adhesin of *E. coli*. The attachment activates signal cascades causing actin rearrangement, which ultimately leads to an internalization of the bacteria into the umbrella cells of the epithelium [7]. The vesicular UPECs can be recognized by the innate immune system within the cells and exported via exocytosis back to the bladder where they are exposed to neutrophils and destroyed. However, UPEC strains employ several strategies to evade the host immune system, which facilitates formation of intracellular bacterial communities (IBCs); this enables bacteria to multiply, mature and infect other cells [27, 28]. Furthermore, this can potentially lead to more severe infection or risk for recurrence as the pathogen might remain hidden inside the uroepithelial cells.

### Treatment

UTIs are commonly treated with antibiotics but due to increasing development of multidrug resistant strains, there is a need for alternative and complementary remedies [29–31]. The most commonly prescribed antibiotics are sulfonamides, trimethoprim, fluoroquinolones, fosfomycin, and beta-lactams, but resistance to these drugs varies between 15 and 50% in Europe, limiting their use for severe infections [32]. The use of some antibiotics, such as amoxicillin, has been restricted for UTI owing to the development of antibiotic resistance [17]. An international study on antibiotic susceptibility patterns performed in 17 European countries including 4734 women with acute uncomplicated UTI showed that 42% of the *E. coli* associated UTIs were resistant to one or more antibiotics. From the 12 used antimicrobials, the resistance was the highest for ampicillin (29.8%) and sulfamethoxazole (29.1%). Antibiotic resistance was relatively common also to trimethoprim (14.8%), trimethoprim/sulfamethoxazole (14.1%) and nalidixic acid (5.4%). Regional differences existed as in Spain and Portugal, antibiotic resistance was higher compared with the Nordic countries and Austria [33]. Another study performed in the US/Canada involving 40 clinical centers showed that *E. coli* resistance to ampicillin was 37.7, 21.3% to trimethoprim/sulfamethoxazole, 5.5% to ciprofloxacin, 5.1% to levofloxacin and 1.1% to nitrofurantoin [34]. Antibiotic resistance of UPEC has also been shown to be a prominent threat in Asia-Pacific regions [35, 36].

The family of Enterobacteriaceae (incl. *E. coli*) has acquired plasmids containing genes for extended-spectrum of β-lactamases (ESBL). β-lactamases cleave the amide bonds of β-lactams, thus the ability to produce β-lactamases compromises the antibiotic treatments making β-lactams ineffective in both uncomplicated and complicated UTIs [37, 38]. Research shows that UPEC strains isolated from the elderly who suffer from rUTIs, are cell-wall deficient i.e. providing to these strains resistance to antibiotics targeting the bacterial cell walls [39]. The WHO has listed Enterobacteriaceae as one of the pathogen groups that should be prioritized for research owing to its resistance patterns specifically to the third generation cephalosporin (β-lactam) that affects UTI treatments [6].

In addition to the development of multi-resistant strains the use of antibiotics for UTI has other disadvantages. For instance, in 25–35% of the cases rUTI occurred within 6 months of the first antibiotic treatment [40, 41] and in 44% of the cases within 12 months [10, 42]. Furthermore, repetitive use of antibiotics disturbs the indigenous microbiota especially in the gastrointestinal tract and vagina, and their use is often associated with unpleasant side effects such as nausea, vomiting, diarrhea, headaches, and skin rash. Thus, a search for alternative approaches to be used especially as a prophylactic in rUTIs is necessitated. Among the most commonly proposed natural alternatives is the daily intake of cranberries and/or D-mannose [31].

## D-mannose

The interest towards D-mannose and UTIs dates back to the 1970s [43, 44]. The emergence of antibiotic resistance related to uropathogens, especially UPEC, has maintained this interest. D-mannose is marketed globally as a dietary supplement and it is mainly targeted for supporting urinary tract health either as a standalone product or combined with cranberry extract or probiotics.

D-mannose (C_6_H_12_O_6_) (mannose) is one of the nine monosaccharides (D-glucose, D-galactose, D-mannose, D-xylose, L-fucose, D-glucuronic acid, N-acetyl-D-glucosamine, N-acetyl-D-galactosamine, and N-acetylneuraminic acid) commonly found in animal glycans and abundant in vertebrate glycoconjugates.

In the human body, D-mannose is primarily synthesized from glucose or is derived from the breakdown of endogenous glycoconjugates. Catabolism of D-mannose occurs via glycolysis after which it is used for energy or incorporated into glycans [45, 46]. D-mannose contributes to the glycoprotein synthesis, more specifically to the glycosylation of certain proteins (post-translational modifications). Many cell types have mannose-specific receptors, hence, stable blood mannose levels are important for facilitating efficient/constant mannose uptake to different cells [47]. Physiological blood D-mannose level varies between 50 to 100 μM [4].

**Fig. 2:**
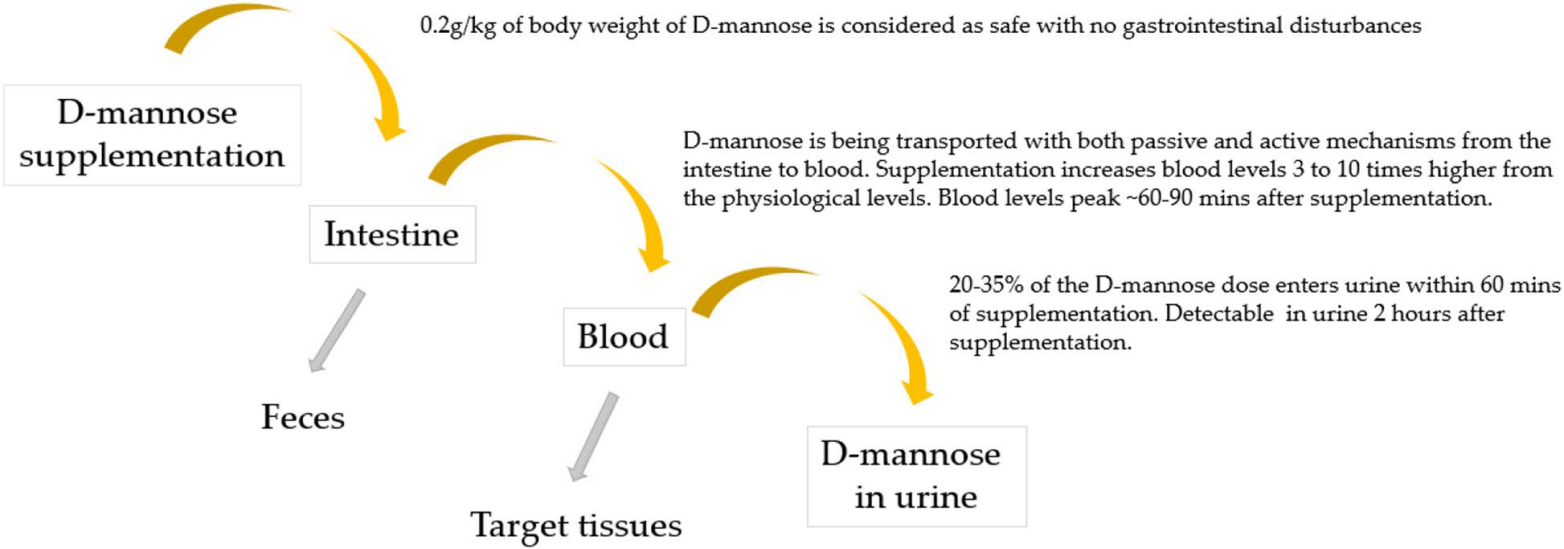
D-mannose, from supplementation to urine. Roughly one third of supplemented D-mannose ends up into urine where it has the potential to block pathogenic *Escherichia coli* from adhering to uroepithelial cells. Some of the D-mannose can be detected in the feces and some is utilized within the target tissues

Fruits such as oranges, apples and peaches contain free D-mannose in relatively small amounts. Furthermore, mannose can be found in the form of galactomannans (undigestible plant polysaccharides) in coffee beans, fenugreek and guar gums [48]. However, the bioavailability of mannose for glycan synthesis in these dietary sources is poor, and likely only partially improved by anaerobic bacteria in the colon [49]. Therefore, dietary mannose is not considered as a significant source of D-mannose for humans. Neverthless, undigestable plant polysaccharides in the colon could lead to other health benefits, for instance via short chain fatty acid production [49], a topic not in the scope of this review. Also yeast cell walls consist of mannans that are undigestible [50]. Further, animal-derived mannose would require specific transport mechanisms. Interestingly, in an animal model of obesity, addition of D-mannose to the diet (at 2%) reduced weight gain, adiposity and liver steatosis and glucose sensitivity. It also led to a change in fecal microbiota with increases in putative beneficial microbes such as *Faecalibaculum* and *Akkermansia* [51]. D-mannose is absorbed into the bloodstream from the gastrointestinal tract after ingestion, the absorption rate being 10% of that of glucose. It is absorbed mainly by passive diffusion across the intestinal barrier, but also active transport molecules have been identified [52]. D-mannose can be administered in dietary supplements in biologically usable forms. Studies indicate that a dose level of 0.2 g/kg of body weight seems to be the upper limit for daily consumption of mannose for a long-term use, as higher doses may cause gastrointestinal disturbances (diarrhea, bloating) [4]. Dietary ingestion increases the blood D-mannose levels 3 to 10-fold from the normal levels in a dose dependent manner [4]. The peak values are reached approximately 60 to 90 min after oral ingestion and return to normal physiological levels after 6 to 8 h the half time being approximately 4 h [4, 53, 54]. A rat study by Alton et al. [47] showed that mannose is relatively fast absorbed (within an hour) from the intestine to the blood, the half time in blood being half an hour. Furthermore, less than 1% of the labeled mannose remained in the intestine, feces and urine after 4–8 h of the gavage, demonstrating the efficacy of mannose uptake from the intestine. Despite the relatively fast increase of D-mannose concentrations in the blood, D-mannose is not fully metabolized in humans. Excess D-mannose (20–35% of the dose) enters urine from the blood circulation within 60 min [45, 46], where it has the potential to interact with mannose-sensitive structures of UPEC and further lowering pathogenic effect of the bacterium. The low renal threshold for mannose (and high for glucose) was demonstrated already by Harding et al. [55] in 1933 in a study where participants were getting a single oral dose of 25 or 50 g of mannose. Mannose supplementation was shown not to affect blood glucose or mannose levels, however, mannose was detected in the urine sample taken 2 h after oral ingestion. Figure 2 describes D-mannose supplementation and its route to urine.

## D-mannose & uropathogenic *E. coli*

### In vitro / preclinical evidence

In vitro and preclinical studies conducted with D-mannose provide insight on potential mechanism of action of D-Mannose against UPEC strains.

As an assumption, sugars like D-mannose, could potentially serve as a carbon source for bacteria and hence induce their growth. However, Scribano and co-workers [56] demonstrated in vitro that D-mannose is not inducing effects on the UPEC metabolism/bacterial growth nor does it interfere with the antibiotic activity. These findings support the suitability of D-mannose in UTI management. Several studies have demonstrated that the binding of *E. coli* FimH to the high-mannose glycoproteins on the surface of urinary tract cells can be inhibited by naturally occurring mannose or designed mannose-derivatives, referred as mannosides [57–60]. The structural analysis by Hung et al. [59] revealed that FimH can envelope mannose molecules in a deep pocket where primarily hydrogen bonds are affecting the complex. Bouckaert et al. [57] demonstrated that the affinity of mannose to FimH is very high, especially compared to other monosaccharides (fructose 15-fold less, glucose 4000-fold less). Animal trials have shown that free D-mannose in urine, even in low concentrations (< 20 μg/ml) can inhibit bacterial adherence mediated by type 1 pili to urinary tract mucosa of pigs [61]. A rat study by Michaels et al.*,* [62] demonstrated that beneficial effects on bacteriuria can be reached already after one day of saccharide injection (D-mannose or D-glucose), the efficacy being dependent on both the injected dose and the amount of *E. coli*. Studies performed in mice, have investigated the potential of small molecular weight FimH antagonists, mannosides, to be used in UTI treatments [63, 64]. Klein et al. [64] demonstrated that orally supplemented FimH antagonist reduced CFU counts of UPEC in the urine by 2 folds and in the bladder of the animals by 4 -fold. Cusumano et al. [63] showed in a murine model of chronic cystitis that orally given active FimH antagonists reduced UPEC colonization in the urethra after 6 h when compared with the control group (phosphate buffered saline). UPEC concentration in the mice treated with antibiotics seemed to be higher than in the mice subject to FimH antagonist. This finding potentially indicates shorter and more effective UTI treatment time by FimH antagonist than with trimethoprim-sulfamethoxazole, an antibiotic. Furthermore, the study showed that IBC formation in the uroepithelium was prevented, supporting the prophylactic potential of the studied mannoside. Although, most of the research has focused on *E. coli* and type 1 pili, it is worthwhile to note that type 1 pili are also found on other bacteria in the Enterobacteriaceae family, such as *K. pneumoniae.* Indeed, in vitro D-mannose has shown potential to inhibit adhesion of a clinical isolate of *K. pneumoniae* [65].

Thus far, immunological effects of D-mannose in the context of UTI are largely unknown. However, a study by Zhang and coworkers [66] suggested that D-mannose has positive immunoregulatory effects on T-cells in mice with autoimmune diabetes and airway inflammation. The role of regulatory T-cells, UTI and D-mannose are worth exploring in further studies.

The affinity between FimH and mannosides shown in vitro and animal models will presumably prevent the bacterial entry and infection of the urinary tract cells and thus provide therapeutic value and scientific rationale for mannose supplementation as a prophylactic treatment for UTIs in humans. In the next section we review and discuss the existing evidence from clinical trials including UTI patients and D-mannose supplementation.

### Clinical evidence of D-mannose in UTI

To identify clinical trials conducted with D-Mannose in UTI, we performed a literature search with terms of “UTI” and “D-mannose” from common databases such as Pubmed, Scopus and Web of Science until January 2021. Original articles were included in this review. The studies meeting the criteria are discussed below and details of the studies are provided in the Tables 1, 2 and 3.

**Table 1:** Clinical trials in acute UTI/rUTIs with treatment supplementations including D-mannose only

Reference	Study Design	Subjects and groups	Supplementation	Main Findings (including safety)
Domenici 2016 [67]	Pilot study, randomized for long-term prophylactic effect	18–65 year old women with acute cystitis and/or history of rUTIs *n* = 43	**Acute:** 13 days; 1.5 g D-mannose twice daily for 3 days and then once a day for 10 days. **Long-term:** 6 months; once a day for a week every other month	D-mannose has potential as an effective agent for both acute UTI and as prophylactic for rUTI in a specific population No AEs
Kranjčec 2014 [68]	Prospective, randomized, open-label, controlled study	18 + years old women with acute cystitis and a history of recurrent cystitis in 3 groups: 1. (*n* = 103) D-mannose 2. (n = 103) Nitrofurantoin 3. (*n* = 102) no prophylaxis *n* = 308	**Long-term:** 6 months once a day D-mannose: 2 g in 200 ml water Nitrofurantoin: 50 mg	D-mannose may be beneficial for UTI prevention. The decreased recurrence rate did not differ between patients who took Nitrofurantoin and D-mannose Mild AEs in 7.8% (diarrhea) of D-mannose group compared to 27.2% (various AEs) in Nitrofurantoin group
Phe 2017 [69]	A single-center, open-label, feasibility study	46–59 year old MS patients using and not using urinary catheters, experiencing rUTIs *n* = 22	**Long-term:** 16 weeks, 1.5 g D-mannose twice a day	D-mannose is safe and feasible supplementation for patients having MS. For efficacy, further studies are needed. No AEs
Porru 2014 [70]	Pilot study, randomized, cross-over trial	22–54 years old female patients with acute symptomatic UTI and ≥ 3rUTIs during the preceding 12 months *n* = 60	**Long-term** cross-over design: Group 1: 1 g D-mannose 3 times a day, every 8 h for 2 weeks, and subsequently 1 g twice a day for 22 weeks. Group 2: 5-day antibiotic therapy with trimethoprim/sulfamethoxazole 160 mg/800 mg twice a day, followed by a single dose at bedtime for 1 week each month in the following 23 weeks Cross-over point at week 24	D-mannose was shown to be effective and safe in preventing rUTIs in women. The proportion of infection free women was greater in D-mannose group compared to antibiotic group. No AEs mentioned

*UTI* urinary tract infection, *rUTI* recurrent urinary tract infection, *AE* adverse event, *MS* multiple sclerosis

**Table 2:** Clinical trials in acute UTI/rUTIs with treatment supplementations including D-mannose and probiotics

Reference	Study Design	Subjects and groups	Supplementation	Main Findings (including safety)
Del Popolo 2018 [71]	Pilot study, non-randomized	68 women and 17 men affected by recurrent symptomatic cystitis. Of those, 33 women and 13 men suffered from neurogenic bladder *n* = 85	**Acute:** 5-days bid 1000 mg of D-mannose, 200 mg of dry willow extract (salicin) (attack phase), followed by 7-days bid with 700 mg of D-mannose plus 50 mg (10^9^ CFU) of *L. acidophilus* La-14 (maintenance treatment). **Long term:** The maintenance treatment was repeated for 15 days every month for the next two months.	Combination treatment was effective in acute UTI and as prophylaxis No significant AEs reported
Milandri 2018 [72]	Single-center, single-arm, uncontrolled observational study	19–87-year-old female patients who underwent urodynamic invasive procedure *n* = 100	**Long-term:** After invasive surgery, 14-days bid 1000 mg D-mannose, 200 mg *H. sabdariffa*, and 10^9^ CFU *L. plantarum* Lp-115	Risk of bacteriuria and UTI in women could be reduced with the studied product No AEs
Murina 2020 [73]	Single-center	Premenopausal women aged 18–50 years with an acute UTI and a history of recurrent uncomplicated UTIs *n* = 55	After 2 days Fosfomycin (3 g once a day) the following combination treatment: Lactoflorene Cist® including 10^9^ CFU *L. paracasei* LC11, cranberry extract and 1000 mg D-mannose: Group 1: once a day for 10 days/month for 90 days (*n* = 19) Group 2: once a day for 90 days (n = 19) Group 3: No treatment (*n* = 17)	Both treatments efficient and safe as prophylaxis for rUTIs. No AEs
Vicariotto 2014 [74]	A pilot prospective study	Premenopausal, nonpregnant women diagnosed with acute uncomplicated cystitis *n* = 33	**Acute:** For 30 days 2 doses a day **Long-term:** after 30 days acute phase, 1 dose a day until day 60 Dose: 2.5 × 10^9^*L. plantarum* LP01 and 1 billion *L. paracasei* LPC09 and *S. thermophilus* ST10, 250 mg of tara gum, 500 mg of a high proanthocyanidins cranberry extract and 250 mg of D-mannose	Significant improvement in the UTI symptoms (dysuria, frequent voiding, urgency, and suprapubic pain) in long-term No AEs mentioned

*UTI* urinary tract infection, *rUTI* recurrent urinary tract infection, *AEs* adverse events, *CFU* colony forming units, *bid* two times a day

**Table 3:** Clinical trials in acute UTI/rUTIs with treatment supplementations including D-mannose in combination with other supplements

Reference	Study Design	Subjects and groups	Supplementation	Main Findings (including safety)
De Leo 2017 [75] Article in Italian	Multicenter, Randomized, controlled trial	40 to 50 year old women suffering from recurrent episodes of cystitis; *n* = 150	1 Kistinox® Forte sachet per day including cranberry (*Vaccinium macrocarpon*), Noxamicina® (propolis extract) and 500 mg D-mannose during the first 10 days of the month, for 3 months (n = 100). No treatment in the control group (*n* = 50)	Product efficient and well-tolerated in treatment of acute UTI and reducing rUTI No AEs
Efros 2010 [76]	Prospective, dose-escalation study	18 to 75 years old women with history of recurrent UTIs (no acute infection) *n* = 28 (planned) *n* = 23 (actual) − 6 per dose group	12 weeks daily dose of 15 ml, 30 ml, 45 ml, 60 ml, 75 ml or 90 ml of UTI-STAT with Proantinox 3875 mg Proantinox (cranberry concentrate [4:1], ascorbic acid, D-mannose, fructo-oligosaccharides, and bromelain) per 30 ml D-mannose dose not indicated	Safe and well tolerated. Efficient in reducing rUTI incidence and increasing quality of life. AES: 9 reported (nausea, heartburn, headache, dyspepsia (4), diarrhea, back pain) Max tolerated dose set for 60 ml/day.
Genovese 2018 [77]	A randomized three-arm parallel group intervention trial	Adult Caucasian females with acute uncomplicated cystitis history of recurrent UTIs *n* = 72	12 weeks with follow-up at 24 weeks. group A: D-mannose 420 mg + berberine, arbutin and birch (*n* = 24) group B: D-mannose 420 mg + berberine, arbutin, birch and forskolin (n = 24) group C: D-mannose 500 mg + proanthocyanidins (n = 24)	Plant-based supplements reduce the risk for UTI but no specific benefits for D-mannose alone No AEs
Manno 2019 [78]	Prospective comparative study	Women with acute cystitis and history of recurrent cystitis *n* = 40	12 weeks including follow-up time **Acute:** Fosfomycin Tromethamine (3 g) single dose (UROFOS®) for all participants **Long-term:** 2 sachets for 2 weeks and one sachet for two additional weeks as follows: group A: UROIAL containing S&R PACs (250 mg) with type-A proanthocyanidins (72 mg), D-mannose (1000 mg), chondroitin sulfate (200 mg), vitamin C (120 mg) and hyaluronic acid (100 mg) (*n* = 20) group B: no treatment (n = 20)	Complete remission in 37 participants after fosfomycin. Lower UTI episodes and symptoms in treatment group after 4 week’s intervention and follow-up time. No AES mentioned
Marchiori 2017 [79]	Observational, retrospective study	Pre- and postmenopausal women who had survived breast cancer and had recurrent cystitis *n* = 60 (50 had reached menopause)	**Long-term:** Group 1 - antibiotic therapy associated with NDM (n = 40) given 12 h after emptying bladder for 60 days followed by dose 24 h after emptying bladder for 4 months, Group 2 - antibiotics alone (n = 20) NDM dose: D-mannose 500 mg, N-acetylcysteine 100 mg and *Morinda citrifolia* fruit extract 200 mg (NDM) Antibiotic options depending on microbial sensitivity: fosfomycin - 3 g per day for two days every 15 days for three cycles, nitrofurantoin - 1cps 100 mg tid for 6 days and ciprofloxacin - 1000 RM or prulifloxacin - 600 mg 1 cps/day for 6 days	Greater efficacy in NDM combined with antibiotic in reducing UTIs and urinary discomfort compared to antibiotics only No AEs related to IP usage specified
Palleschi 2017 [80]	Prospective, randomized study	~ 65.4 years old male [42] and female [38] patients eligible for urodynamic examination. *n* = 80	**Acute preventive** Group A: antibiotic Prulifloxacine 400 mg/day for 5 days (n = 40), Group B: D-mannose 500 mg, N-acetylcysteine 100 mg and *Morinda citrifolia* fruit extract 300 mg, twice a day for 7 days (n = 40)	D-mannose and NAC therapy resulted similar results to the antibiotic therapy in preventing UTIs in patients submitted to urodynamic examination. Considered as usable alternative treatment No AEs
Panchev 2012 [81] Article in Bulgarian	Multicenter, comparative, observational study	Female patients with acute uncomplicated urinary bladder infections (Age not reported) *n* = 158	**Acute:** Group 1: Product containing D-mannose 1000 mg, standardized dry birch leaf extract 50 mg, standardized dry cranberry extract 50 mg according to manufacturer’s instructions (*n* = 86) Group 2: Ciprofloxacin 500 mg twice daily for 3 days (n = 72)	Better effectiveness related to symptoms and clinical outcomes with the product compared to antibiotic was reported No AEs
Rădulescu 2020 [82]	a pilot, randomized study	non-pregnant, healthy women with uncomplicated lower UTI Age range 18–60 years *n* = 93	**First phase/Acute:** 1) Antibiotic (TMP-SMX) (*n* = 45) or 2) Antibiotic + D-mannose (1000 mg) + cranberry (400 mg) (Uro-Care with CranActin®)(*n* = 48) for 7 days **Second phase/ prophylaxis:** For cured participants either 1) D-mannose + cranberry (*n* = 47) or 2) placebo (*n* = 46) for 21 days	Higher cure rate after acute phase in the combined group especially in the resistant strains. No significant differences between the active and the placebo in the second phase of the study No AEs related to IP usage specified
Russo 2020 [83]	A prospective, randomized, no-placebo, controlled study	~ 67.2 years old postmenopausal women undergoing surgery for cystocele n = 40	Active: cranberry, D-mannose, Boswellia, Curcuma and Noxamicine VR (Kistinox ActVR) twice a day for 2 weeks starting from surgery (n = 20) Control: only surgery (n = 20)	Symptom relief was reported in the active group compared to control. No differences in UTI incidences No AEs
Salinas-Casado 2018 [84] Article in Spanish	A multicenter, double-blind, randomized, experimental study	~ 48 years old women with non-complicated UTI *n* = 95	**Long-term:** Group 1: 2 g of D-mannose, 140 mg of PAC and 7.98 mg of ursolic acid together with vitamins A, C and E, and the Zinc trace element (Manosar®) (*n* = 44) once a day for 24 weeks Group 2: 240 mg proanthocyanidins (*n* = 51) as a single dose/day	Product was reported to be more efficient for preventing rUTI than single dose of PAC AEs: 21.4% in Group 1 and 21.6% in Group 2 (diarrhea, headache, vaginal discomfort, nausea rash)
Salinas-Casado 2020 [85] Article in Spanish	A multicenter, randomized and double-blind experimental study	~ 49.5 years old women with a history of recurrent UTIs *n* = 184	Group1: 2 g of D-mannose, 140 mg of PAC and 7.98 mg of ursolic acid together with vitamins A, C and E, and the Zinc trace element (Manosar®) (*n* = 90) once a day for 24 weeks Group 2: 240 mg proanthocyanidins (*n* = 94) as a single dose	Product was reported to be more efficient for preventing rUTI than single dose of PAC AEs: 16.8% of participants experienced AEs (12 in Group 1 and 19 in Group 2) (diarrhea, headache, vaginal discomfort, nausea rash)

*UTI* urinary tract infection, *rUTI* recurrent urinary tract infection, *AEs* adverse events, *cps *capsule, *tid* three times a day, *IP* investigational product, *NDM* N-acetylcysteine D-mannose *Morinda citrifolia*, *PAC* proanthocyanidin

#### Acute and long-term effects of D-mannose in UTI

Several studies have investigated both acute and prophylactic effects of D-mannose, or more often, D-mannose combined with antibiotic or other alternative supplements, in UTI. These studies have focused mostly on females suffering from acute or rUTIs.

To date, four studies have assessed the effect of supplementation, including D-mannose only, in UTIs (Table 1). A pilot study by Domenici and co-authors showed that D-mannose could be used for acute UTI (13 days treatment) but also has potential as a prophylaxis (6 months treatment) in women with symptomatic (dysuria, frequency, urgency, supra-pubic pain, nocturia, and hematuria) or asymptomatic UTI (diagnosed as ≥10^3^ CFU/mL of urine) [67]. Most of the symptoms were shown to decrease significantly compared to control group. There was also a statistically significant difference in the rUTI percentages between the active and control groups (4.5 and 33.3%, respectively). In an open-label clinical trial by Kranjčec et al. [68], adult women with acute UTI and tendency for recurrence consumed either D-mannose, nitrofurantoin or no prophylaxis for 6 months after acute antibiotic treatment. The risk for rUTIs decreased significantly in both prophylactic treatments. There were no differences between the study groups receiving either D-mannose or antibiotic, suggesting that D-mannose is as effective as antibiotics to be used as an alternative treatment in preventing rUTIs. An open-label, feasibility study including multiple sclerosis patients demonstrated that a 16-weeks daily oral supplementation with D-mannose significantly reduced the number of UTIs (by 75% in patients without urinary catheter and by 63% in those with catheter) [69]. A cross-over study in adult women demonstrated that D-mannose supplementation delays significantly the onset of rUTI compared to antibiotics [70]. In the study, the recurrence of UTI occurred on average in 200 days with daily oral supplementation of D-mannose, whereas for used antibiotic the time to recurrent infection was on average 52.7 days.

D-mannose’s effect on UTI/rUTIs has also been studied in combination with probiotics (Table 2). Del Popolo et al. [71] demonstrated in a pilot, open-label study in women (*n* = 68) and men (*n* = 17) including both non-neurological and neurological patients, that an oral combination of D-mannose and salicin, for acute UTI, together with *Lactobacillus acidophilus* La-14 for maintenance/prevention, is a promising approach for rUTIs. The acute treatment consisted of 5-day supplementation of D-mannose + salicin 3 times a day and the maintenance treatment 7-days with D-mannose + *L. acidophilus* La-14 (1 × 10^9^ CFU) twice a day. The UTI symptoms improved significantly after the acute treatment (2 weeks), long-term treatment (12 weeks = end of treatment) and also 1 month after the supplementation had ended, compared to the baseline symptoms. An observational study by Milandri et al. [72] demonstrated that 14-day phytotherapeutic supplementation including D-mannose, *Hibiscus sabdariffa*, and *Lactiplantibacillus plantarum* Lp-115 after urodynamic procedure can reduce the risk of bacteriuria and UTI in women. A study by Murina et al. [73] investigated UTI patients in a controlled trial. After a 2-day treatment with antibiotics and confirming that patients were free of symptoms, they received *Lacticaseibacillus paracasei* LC11, cranberry and D-mannose for the 10 first days of 3 months (Group 1) or once a day for 90 days (Group 2) or no treatment at all (Group 3). In the study 87.7% of patients in the Group 1 remained free of UTI until day 90 and 65.8% of patients were not diagnosed with UTI at day 150. In the Group 2 the 84.9% were UTI free at day 90 and 68.8% at day 150, whereas in the Group 3 (control) 42% at day 90 and 36.9% at day 150 were UTI free. These results showed that in both active treatment groups, UTI recurrence was significantly lower compared to the control group during the 150 days trial. There was no significant difference in the recurrence frequencies between the two treatment types i.e. whether the treatment was continuous or happened only for 10 days each month. Another study suggested that the supplementation including cranberry, D-mannose and tara gum in addition with probiotic strains *L. plantarum* LP01 (2.5 × 10^9^ CFU), *L. paracasei* LPC09 (10^9^ CFU) and *Streptococcus thermophilus* ST10 (10^9^ CFU) relieved the symptoms of acute UTI [74]. The symptom relief was detectable 1 month after starting the treatment (2 doses daily) and remained when supplementing one dose daily for an additional month (Day 60) and 1 month after the treatment had ended (Day 90).

Most commonly in UTI studies, D-mannose is combined with other plant-based supplements (Table 3). A randomized study on peri- and postmenopausal women with rUTI showed that oral supplementation of a product containing cranberry, propolis extract and D-mannose, was well-tolerated and effective in UTI treatment and in reducing risk for rUTIs [75]. In the studied treatment group, the product was administered for 10 days at the beginning of each month for 3 months, whereas control group did not receive any treatment. The urinary symptoms were shown to be completely alleviated from 92 of the studied women. A randomized study by Genovese et al. [77] in UTI patients investigated the effects of oral D-mannose and different botanicals for 12 weeks on UTI recurrence. rUTI diagnosis was assessed by microbial analyses from urine samples, vaginal swabs and vaginal smear. The study demonstrated that either D-mannose together with berberin, arbutin, birch or D-mannose together with berberin, arbutin, birch, and forskolin were more effective in preventing rUTIs than D-mannose in combination with proanthocyanidin emphasizing the beneficial effects of combination of various plant-based supplements in lowering the risk for rUTIs. Manno et al. [78] hypothesized that efficacy of D-mannose + cranberry as a prophylaxis for rUTI could be enhanced by adding hyaluronic acid, and chondroitin sulfate into the study product. Adults with acute cystitis were treated with single dose of antibiotics after which they were randomized to either treatment or control group. Patients consumed daily 2 sachets of the study product for 2 weeks followed by one sachet for another two weeks. After 12 weeks of follow-up, symptoms were relieved in 85% of participants who had consumed the combination product, whereas the symptoms were relieved only in 10% of participants in the control group. Bacterial counts revealed that *E. coli* was detected from the urine of 1 patient in the treatment group after 12 weeks, compared to that of 10 in the control (Baseline numbers 15/20 and 16/19, respectively). An observational study performed on 60 female breast cancer survivors indicated, that a combination of oral antibiotic with D-mannose, N-acetylcysteine and *Morinda citrifolia* fruit extract provided more benefits by reducing UTIs and urinary discomfort when compared to antibiotics-only in a study which lasted for 6 months [79]. The same product was used in a larger population including 42 men and 38 women submitted to urodynamic investigation [80]. This randomized study showed that there were no differences in UTI recurrences between the group using antibiotics and the group using nutraceutical agents, indicating that a product containing D-mannose, N-acetylcysteine and *Morinda citrifolia* fruit extract could be a potential prophylaxis alternative for UTI in this group of patients. Panchev et al. [81] assessed the efficacy of an oral combination product containing D-mannose, birch leaf, and cranberry extract on acute UTI in an observational study. The study results showed that after 3-day supplementation the clinical - and symptomatic improvements were faster with the D-mannose containing investigational product (IP) compared to antibiotics (mean time being 24 h and 46 h, respectively). At 48 h, 97% of the IP group had improved symptoms, whereas only 65.3% in the antibiotic group. A pilot study by Radulesku et al.*,* [82] showed that cure rate in acute UTI was higher when combining 7 days antibiotic treatment with an oral IP containing D-mannose and cranberry (84.44% in the antibiotic alone and 91.66% in the antibiotic + IP) – though not reaching statistically significant difference between the groups. When looking at only the patients with antibiotic resistant strains, the cure rate was significantly better in the combined group. The cure rate was also assessed after 21 days prophylactic treatment with the IP (no antibiotic involved), showing no significant differences between the IP and placebo. The potential beneficial effect of 2 weeks cranberry, D-mannose, *Boswellia*, *Curcuma* and Noxamicine supplementation on perceived lower urinary tract symptoms after cystocele operation was assessed by Russo et al. [83] in a randomized trial. In the study, postmenopausal women received supplementation twice a day for two weeks starting on the operation day or operation only. Specific symptom scores from the used questionnaire were reported to be lower in the group receiving the supplementation. However, no differences in the perioperative outcomes or UTI incidences were detected between the study groups during the follow-up. The recurrence of UTIs was also assessed in a randomized double-blind study in adult women [84]. The study products were 1) a food supplement containing D-mannose, proanthocyanidins, ursolic acid and vitamins A, C and E, and zinc and 2) a compound containing proanthocyanidins (polyphenols). In the study, once a day consumption for 24 weeks of the investigational product containing D-mannose was more effective in lowering the risk for UTI than a single daily dose of proanthocyanidin. A similar study with a larger study population was performed with similar results [85].

### Safety of supplemented D-mannose

Despite the potential benefits of D-mannose in UTI, some mice studies have shown that prenatal mannose supplementation causes embryonic lethality and eye defects among the mice who survived [86]. In this trial, the dose ranged from 1 to 5% in the drinking water. In humans, safety and tolerability of a D-mannose containing product has been tested in a so called maximal tolerated dose design study [76]. This study showed that the product containing D-mannose was well-tolerated up to 90 ml of study product (D-mannose amount not specified). Of note, the main ingredient in the product was cranberry liquid. In the above reviewed clinical trials where D-mannose was investigated as a single active ingredient with a daily dose between 2 and 3 g [67–70], no serious adverse events were associated with the use of D-mannose. In addition, a systematic review and meta-analysis by Lenger et al. [87] concluded that D-mannose was well tolerated with minimal side effects—only a small percentage experiencing diarrhea. The occurrence of adverse events is likely to be dose-depended as daily doses exceeding 0.2 g/kg of body weight may cause diarrhea and bloating [4]. Of note, in human diabetics, blood glucose balance could potentially be disturbed by mannose supplementation [50]. This should be taken into account when considering D-mannose supplementation among diabetics and pregnant women.

## Discussion

While antibiotics are still the mainstay for treatment of acute UTI, their use as prophylaxis has already led to the development of resistant bacterial strains; compromising treatments, and accumulating challenges over time. Furthermore, the antibiotic side effects can cause discomfort and predispose patients to other infections.

It has been demonstrated in both animal and human studies that the renal threshold for D-mannose is low i.e. excess D-mannose is secreted into urine [45, 46, 55]. In addition, good affinity of mannose and mannosides to *E.coli* type 1 pilus structures has been shown by several in vitro experiments [57–60]. Furthermore, based on an animal trial even at concentrations as low as 20 μg/ml, D-mannose can efficiently block uropathogenic *E. coli* adhesion to the urinary tract, subsequently lowering the risk for UTI [61]. Several clinical trials have assessed the potential of D-mannose supplementation to improve either acute clinical and symptomatic outcome of UTI or/and shorten the time-to-relapse in rUTIs. To date, altogether 19 peer-reviewed clinical trials have been published (Tables 1, 2 and 3). However, only four studies were conducted with D-mannose alone, from which 2 trials [67, 70] assessed both acute and long-term preventive effect of D-mannose on UTI and 2 trials [68, 69] only the preventive effect. In most of the studies (*n* = 15) a combined effect of D-mannose and other “nutraceutical(s)”, such as cranberry extract or probiotic, was studied. In addition, there are few studies comparing the efficacy of D-mannose supplementation and antibiotics on treatment of acute UTI or as prophylaxis. From the 19 studies reported here, 18 indicate that D-mannose supplementation, alone or combined with other products, could be beneficial in the management of UTI; one study [69] reported on feasibility and not efficacy. Of these 18 studies, seven report on treatment of acute UTI; six report on beneficial effects and one [83] did not observe a difference in UTI with the control group but did nevertheless observe a reduction in symptoms. Further, 14 of the 18 studies reported on prophylaxis in the management of rUTI. Of these 14 studies, 13 reported on reductions in rUTI, one study [82] did not report a difference in recurrence compared to the antibiotic control. Thus, D-mannose may help to improve clinical/symptomatic recovery rate from UTI - sometimes even faster than some of the used antibiotics – and/or may especially have potential as a prophylactic by decreasing the risk for rUTIs. However, to date no common guidelines for the D-mannose treatment duration, dose and combination exist. Furthermore, no health claims thus far have been approved for the use of D-mannose in UTI in any jurisdiction. Such claim would protect the consumer seeking self-help and provide health-care professionals with confidence to recommend D-mannose as an alternative or complementary treatment.

rUTI is a common challenge especially among women [88]. Imbalance of the urogenital bacteria caused by frequent intercourse (especially younger women) or postmenopausal age are risk factors for UTI occurrence. Frequent infections and the use of antibiotics lead to changes in the microbiota in the urogenital area. Especially antibiotic use may affect the dominance of indigenous lactobacilli, and potentially creating suitable environment for the uropathogens to thrive. Therefore, also the use of probiotic lactobacilli to reduce the risk of rUTIs by supporting vaginal and urinary microbiota has gained attention. Currently (mid-2021), four clinical trials including both D-mannose and probiotics (one had also cranberry) have been conducted showing promising outcomes related UTI symptoms and reoccurrence rates.

Of the eight studies registered in clinicaltrials.gov (accessed 3rd February 2022) investigating the effect of D-mannose on UTI, only one has results, but these seem not to have been published in the scientific literature while another study has an ‘unknown’ status. Further, one study was terminated and the remaining five studies are in various stages of recruitment. Thus, although more results are to be expected in the future, it also highlights the challenge of potential reporting bias. This is especially challenging when only a limited number of studies are available as in the case of D-mannose and UTI.

## Conclusions

In addition to female gender, sexual activity at young age and higher age in general, specific conditions such as diabetes, neurologic conditions, chronic institutional residence, and chronic urinary catheterization might predispose to rUTIs. Therefore, individuals in need of repetitive antibiotic treatments, going through urogenital procedures or women with changed bacterial environment in the urogenital area would benefit the most from a non-antibiotic alternative. Due to increasing antibiotic resistance among UTI pathogens, the burden caused by UTIs is expected to increase creating a high demand for alternative options. For the treatment of acute UTI, antibiotics are likely to remain the first choice. Supplementing antibiotics with D-mannose may increase treatment success. However, for prophylaxis in reducing rUTI, D-mannose appears to have great potential with minimal side effects. The overall picture of preclinical and clinical studies with D-mannose in the management of UTI is favorable, as discussed here and in a recent narrative review by De Nunzio et al. [89]. D-mannose has also been shown to be relatively safe and well-tolerated. Yet, the quality of these studies leaves something to be desired; they are mostly confounded with other active ingredients, have small numbers of participants, are open label or uncontrolled. What is first and foremost needed are sufficiently powered, well-designed double-blinded, randomized, and placebo-controlled clinical trials with solely D-mannose in the active product; distinguishing between treatment and prophylaxis. Such studies are registered in clinicaltrials.gov; we look forward to their results.

## Data Availability

Not applicable.

## References

[1] Beerepoot MA, ter Riet G, Nys S, van der Wal WM, de Borgie CA, de Reijke TM (2011). Cranberries vs antibiotics to prevent urinary tract infections: a randomized double-blind noninferiority trial in premenopausal women. Arch Intern Med.

[2] Khandelwal P, Abraham SN, Apodaca G (2009). Cell biology and physiology of the uroepithelium. Am J Physiol Ren Physiol.

[3] Xie B, Zhou G, Chan SY, Shapiro E, Kong XP, Wu XR (2006). Distinct glycan structures of uroplakins Ia and Ib: structural basis for the selective binding of FimH adhesin to uroplakin Ia. J Biol Chem.

[4] Alton G, Kjaergaard S, Etchison JR, Skovby F, Freeze HH (1997). Oral ingestion of mannose elevates blood mannose levels: a first step toward a potential therapy for carbohydrate-deficient glycoprotein syndrome type I. Biochem Mol Med.

[5] Scaglione F, Musazzi UM, Minghetti P (2021). Considerations on D-mannose mechanism of action and consequent classification of marketed healthcare products. Front Pharmacol.

[6] Organization. WH (2017). Prioritization of pathogens to guide discovery, research and development of new antibiotics for drug-resistant bacterial infections, including tuberculosis.

[7] Flores-Mireles AL, Walker JN, Caparon M, Hultgren SJ (2015). Urinary tract infections: epidemiology, mechanisms of infection and treatment options. Nat Rev Microbiol.

[8] Stamm WE, Norrby SR (2001). Urinary tract infections: disease panorama and challenges. J Infect Dis.

[9] Foxman B, Brown P (2003). Epidemiology of urinary tract infections: transmission and risk factors, incidence, and costs. Infect Dis Clin N Am.

[10] Foxman B (2014). Urinary tract infection syndromes: occurrence, recurrence, bacteriology, risk factors, and disease burden. Infect Dis Clin N Am.

[11] de Lastours V, Foxman B (2014). Urinary tract infection in diabetes: epidemiologic considerations. Curr Infect Dis Rep.

[12] Patterson JE, Andriole VT (1997). Bacterial urinary tract infections in diabetes. Infect Dis Clin N Am.

[13] Sewify M, Nair S, Warsame S, Murad M, Alhubail A, Behbehani K (2016). Prevalence of urinary tract infection and antimicrobial susceptibility among diabetic patients with controlled and uncontrolled Glycemia in Kuwait. J Diabetes Res.

[14] Shuman EK, Chenoweth CE (2010). Recognition and prevention of healthcare-associated urinary tract infections in the intensive care unit. Crit Care Med.

[15] Lee EA, Malatt C (2011). Making the hospital safer for older adult patients: a focus on the indwelling urinary catheter. Perm J.

[16] Medina M, Castillo-Pino E (2019). An introduction to the epidemiology and burden of urinary tract infections. Ther Adv Urol.

[17] Schmiemann G, Kniehl E, Gebhardt K, Matejczyk MM, Hummers-Pradier E (2010). The diagnosis of urinary tract infection: a systematic review. Dtsch Arztebl Int.

[18] Brubaker L, Putonti C, Dong Q, Wolfe AJ. The human urobiome. Mamm Genome. 2021;32:232–8. 10.1007/s00335-021-09862-8 33651197

[19] Ceprnja M, Oros D, Melvan E, Svetlicic E, Skrlin J, Barisic K (2021). Modeling of urinary microbiota associated with cystitis. Front Cell Infect Microbiol.

[20] Price TK, Hilt EE, Thomas-White K, Mueller ER, Wolfe AJ, Brubaker L (2020). The urobiome of continent adult women: a cross-sectional study. BJOG..

[21] Wolfe AJ, Brubaker L (2019). Urobiome updates: advances in urinary microbiome research. Nat Rev Urol.

[22] Komesu YM, Dinwiddie DL, Richter HE, Lukacz ES, Sung VW, Siddiqui NY (2020). Defining the relationship between vaginal and urinary microbiomes. Am J Obstet Gynecol.

[23] Ammitzboll N, Bau BPJ, Bundgaard-Nielsen C, Villadsen AB, Jensen AM, Leutscher PDC (2021). Pre- and postmenopausal women have different core urinary microbiota. Sci Rep.

[24] Stapleton AE. The vaginal microbiota and urinary tract infection. Microbiol Spectr. 2016;4(6):10.1128/microbiolspec.UTI-0025-2016. 10.1128/microbiolspec.UTI-0025-2016 PMC5746606 28087949

[25] Mestrovic T, Matijasic M, Peric M, Cipcic Paljetak H, Baresic A, Verbanac D. The role of gut, vaginal, and urinary microbiome in urinary tract infections: from bench to bedside. Diagnostics (Basel). 2021;11:7. 10.3390/diagnostics11010007 PMC7822161 33375202

[26] Moreno E, Andreu A, Pérez T, Sabaté M, Johnson JR, Prats G (2006). Relationship between *Escherichia coli* strains causing urinary tract infection. Epidemiol Infect.

[27] Anderson GG, Palermo JJ, Schilling JD, Roth R, Heuser J, Hultgren SJ (2003). Intracellular bacterial biofilm-like pods in urinary tract infections. Science..

[28] Olson PD, Hunstad DA. Subversion of host innate immunity by Uropathogenic Escherichia coli. Pathogens. 2016;5:2. 10.3390/pathogens5010002 PMC4810123 26742078

[29] Gupta K, Hooton TM, Miller L, Uncomplicated UTIIGC (2011). Managing uncomplicated urinary tract infection--making sense out of resistance data. Clin Infect Dis.

[30] Gupta K, Hooton TM, Naber KG, Wullt B, Colgan R, Miller LG (2011). International clinical practice guidelines for the treatment of acute uncomplicated cystitis and pyelonephritis in women: a 2010 update by the Infectious Diseases Society of America and the European Society for Microbiology and Infectious Diseases. Clin Infect Dis.

[31] Loubet P, Ranfaing J, Dinh A, Dunyach-Remy C, Bernard L, Bruyere F (2020). Alternative therapeutic options to antibiotics for the treatment of urinary tract infections. Front Microbiol.

[32] Heisig P (2010). Urinary tract infections and antibiotic resistance. Urologe A.

[33] Kahlmeter G, Eco.Sens. (2003). An international survey of the antimicrobial susceptibility of pathogens from uncomplicated urinary tract infections: the ECO.SENS project. J Antimicrob Chemother.

[34] Zhanel GG, Hisanaga TL, Laing NM, DeCorby MR, Nichol KA, Weshnoweski B (2006). Antibiotic resistance in Escherichia coli outpatient urinary isolates: final results from the north American urinary tract infection collaborative Alliance (NAUTICA). Int J Antimicrob Agents.

[35] Akram M, Shahid M, Khan AU (2007). Etiology and antibiotic resistance patterns of community-acquired urinary tract infections in J N M C hospital Aligarh, India. Ann Clin Microbiol Antimicrob.

[36] Lu PL, Liu YC, Toh HS, Lee YL, Liu YM, Ho CM (2012). Epidemiology and antimicrobial susceptibility profiles of gram-negative bacteria causing urinary tract infections in the Asia-Pacific region: 2009-2010 results from the study for monitoring antimicrobial resistance trends (SMART). Int J Antimicrob Agents.

[37] de Cueto M, Aliaga L, Alos JI, Canut A, Los-Arcos I, Martinez JA (2017). Executive summary of the diagnosis and treatment of urinary tract infection: guidelines of the Spanish Society of Clinical Microbiology and Infectious Diseases (SEIMC). Enferm Infecc Microbiol Clin.

[38] Gupta K, Bhadelia N (2014). Management of urinary tract infections from multidrug-resistant organisms. Infect Dis Clin N Am.

[39] Mickiewicz KM, Kawai Y, Drage L, Gomes MC, Davison F, Pickard R (2019). Possible role of L-form switching in recurrent urinary tract infection. Nat Commun.

[40] Foxman B. Recurring urinary tract infection: incidence and risk factors. Am J Public Health 1990;80(3):331–3. 10.2105/ajph.80.3.331 PMC1404686 2305919

[41] Mabeck CE (1972). Treatment of uncomplicated urinary tract infection in non-pregnant women. Postgrad Med J.

[42] Ikaheimo R, Siitonen A, Heiskanen T, Karkkainen U, Kuosmanen P, Lipponen P (1996). Recurrence of urinary tract infection in a primary care setting: analysis of a 1-year follow-up of 179 women. Clin Infect Dis.

[43] Ballou CE, Lipke PN, Raschke WC (1974). Structure and immunochemistry of the cell wall mannans from Saccharomyces chevalieri, Saccharomyces italicus, Saccharomyces diastaticus, and Saccharomyces carlsbergensis. J Bacteriol.

[44] Spencer JF, Gorin PA (1973). Mannose-containing polysaccharides of yeasts. Biotechnol Bioeng.

[45] Ganda OP, Soeldner JS, Gleason RE, Cleator IG, Reynolds C (1979). Metabolic effects of glucose, mannose, galactose, and fructose in man. J Clin Endocrinol Metab.

[46] Wood FC, Cahill GF (1963). Mannose Utilization in Man. J Clin Invest.

[47] Alton G, Hasilik M, Niehues R, Panneerselvam K, Etchison JR, Fana F (1998). Direct utilization of mannose for mammalian glycoprotein biosynthesis. Glycobiology..

[48] Srivastava M, Kapoor VP (2005). Seed galactomannans: an overview. Chem Biodivers.

[49] Yamabhai M, Sak-Ubol S, Srila W, Haltrich D (2016). Mannan biotechnology: from biofuels to health. Crit Rev Biotechnol.

[50] Sharma V, Ichikawa M, Freeze HH (2014). Mannose metabolism: more than meets the eye. Biochem Biophys Res Commun.

[51] Sharma V, Smolin J, Nayak J, Ayala JE, Scott DA, Peterson SN (2018). Mannose alters gut microbiome, prevents diet-induced obesity, and improves host metabolism. Cell Rep.

[52] Freeze HH, Elbein AD. Glycosylation Precursors. In: Varki A, Cummings RD, Esko JD, Freeze HH, Stanley P, Bertozzi CR, et al., editors. Essentials of Glycobiology. Cold Spring Harbor (NY); 2009. p. 339–72.

[53] Harms HK, Zimmer KP, Kurnik K, Bertele-Harms RM, Weidinger S, Reiter K (2002). Oral mannose therapy persistently corrects the severe clinical symptoms and biochemical abnormalities of phosphomannose isomerase deficiency. Acta Paediatr.

[54] Westphal V, Kjaergaard S, Davis JA, Peterson SM, Skovby F, Freeze HH (2001). Genetic and metabolic analysis of the first adult with congenital disorder of glycosylation type Ib: long-term outcome and effects of mannose supplementation. Mol Genet Metab.

[55] Harding VJ, Nicholson TF, Armstrong AR (1933). Cutaneous blood-sugar curves after the administration of fructose, mannose and xylose. Biochem J.

[56] Scribano D, Sarshar M, Prezioso C, Lucarelli M, Angeloni A, Zagaglia C, et al. D-mannose treatment neither affects Uropathogenic Escherichia coli properties nor induces stable FimH modifications. Molecules. 2020;25(2):316. 10.3390/molecules25020316 PMC7024335 31941080

[57] Bouckaert J, Berglund J, Schembri M, De Genst E, Cools L, Wuhrer M (2005). Receptor binding studies disclose a novel class of high-affinity inhibitors of the Escherichia coli FimH adhesin. Mol Microbiol.

[58] Han Z, Pinkner JS, Ford B, Obermann R, Nolan W, Wildman SA (2010). Structure-based drug design and optimization of mannoside bacterial FimH antagonists. J Med Chem.

[59] Hung CS, Bouckaert J, Hung D, Pinkner J, Widberg C, DeFusco A (2002). Structural basis of tropism of Escherichia coli to the bladder during urinary tract infection. Mol Microbiol.

[60] Old DC (1972). Inhibition of the interaction between fimbrial haemagglutinins and erythrocytes by D-mannose and other carbohydrates. J Gen Microbiol.

[61] Toyota S, Fukushi Y, Katoh S, Orikasa S, Suzuki Y (1989). Anti-bacterial defense mechanism of the urinary bladder. Role of mannose in urine. Nihon Hinyokika Gakkai Zasshi.

[62] Michaels EK, Chmiel JS, Plotkin BJ, Schaeffer AJ (1983). Effect of D-mannose and D-glucose on Escherichia coli bacteriuria in rats. Urol Res.

[63] Cusumano CK, Pinkner JS, Han Z, Greene SE, Ford BA, Crowley JR (2011). Treatment and prevention of urinary tract infection with orally active FimH inhibitors. Sci Transl Med.

[64] Klein T, Abgottspon D, Wittwer M, Rabbani S, Herold J, Jiang X (2010). FimH antagonists for the oral treatment of urinary tract infections: from design and synthesis to in vitro and in vivo evaluation. J Med Chem.

[65] Kil KS, Darouiche RO, Hull RA, Mansouri MD, Musher DM (1997). Identification of a Klebsiella pneumoniae strain associated with nosocomial urinary tract infection. J Clin Microbiol.

[66] Zhang D, Chia C, Jiao X, Jin W, Kasagi S, Wu R (2017). D-mannose induces regulatory T cells and suppresses immunopathology. Nat Med.

[67] Domenici L, Monti M, Bracchi C, Giorgini M, Colagiovanni V, Muzii L (2016). D-mannose: a promising support for acute urinary tract infections in women. A pilot study. Eur Rev Med Pharmacol Sci.

[68] Kranjcec B, Papes D, Altarac S (2014). D-mannose powder for prophylaxis of recurrent urinary tract infections in women: a randomized clinical trial. World J Urol.

[69] Phe V, Pakzad M, Haslam C, Gonzales G, Curtis C, Porter B (2017). Open label feasibility study evaluating D-mannose combined with home-based monitoring of suspected urinary tract infections in patients with multiple sclerosis. Neurourol Urodyn.

[70] Porru D, Parmigiani A, Tinelli C, Barletta D, Choussos D, Di Franco C (2014). Oral D-mannose in recurrent urinarytract infections in women: a pilot study. J Clin Urol.

[71] Del Popolo G, Nelli F (2018). Recurrent bacterial symptomatic cystitis: a pilot study on a new natural option for treatment. Arch Ital Urol Androl.

[72] Milandri R, Maltagliati M, Bocchialini T, Del Prete C, Bianchi G, Rocco BM (2019). Effectiveness of D-mannose, Hibiscus sabdariffa and Lactobacillus plantarum therapy in prevention of infectious events following urodynamic study. Urologia..

[73] Murina F, Vicariotto F, Lubrano C (2021). Efficacy of an orally administered combination of Lactobacillus paracasei LC11, cranberry and D-mannose for the prevention of uncomplicated, recurrent urinary tract infections in women. Urologia..

[74] Vicariotto F (2014). Effectiveness of an association of a cranberry dry extract, D-mannose, and the two microorganisms Lactobacillus plantarum LP01 and Lactobacillus paracasei LPC09 in women affected by cystitis: a pilot study. J Clin Gastroenterol.

[75] De Leo V, Cappelli V, Massaro MG, Tosti C, Morgante G (2017). Evaluation of the effects of a natural dietary supplement with cranberry, Noxamicina(R) and D-mannose in recurrent urinary infections in perimenopausal women. Minerva Ginecol.

[76] Efros M, Bromberg W, Cossu L, Nakeleski E, Katz AE (2010). Novel concentrated cranberry liquid blend, UTI-STAT with Proantinox, might help prevent recurrent urinary tract infections in women. Urology..

[77] Genovese C, Davinelli S, Mangano K, Tempera G, Nicolosi D, Corsello S (2018). Effects of a new combination of plant extracts plus d-mannose for the management of uncomplicated recurrent urinary tract infections. J Chemother.

[78] Manno S, Cicione A, Dell’Atti L, Capretti C, Scarcella S, Cantiello F (2019). Effects of a new combination of cranberry extracts, D-mannose and GAGs for the Management of Uncomplicated Urinary Tract Infection. Endocrinol Diabetes Metab J.

[79] Marchiori D, Zanello PP (2017). Efficacy of N-acetylcysteine, D-mannose and Morinda citrifolia to treat recurrent cystitis in breast Cancer survivals. In Vivo.

[80] Palleschi G, Carbone A, Zanello PP, Mele R, Leto A, Fuschi A (2017). Prospective study to compare antibiosis versus the association of N-acetylcysteine, D-mannose and Morinda citrifolia fruit extract in preventing urinary tract infections in patients submitted to urodynamic investigation. Arch Ital Urol Androl.

[81] Panchev P, Slavov C, Mladenov D, Georgiev M, Yanev K, Paskalev E (2012). A multicenter comparative observation on the effectiveness and the rapidness of the effect of Cystostop rapid versus antibiotic therapy in patients with uncomplicated cystitis. Akush Ginekol (Sofiia).

[82] Radulescu D, David C, Turcu FL, Spataru DM, Popescu P, Vacaroiu IA (2020). Combination of cranberry extract and D-mannose - possible enhancer of uropathogen sensitivity to antibiotics in acute therapy of urinary tract infections: results of a pilot study. Exp Ther Med.

[83] Russo E, Montt Guevara M, Giannini A, Mannella P, Palla G, Caretto M (2020). Cranberry, D-mannose and anti-inflammatory agents prevent lower urinary tract symptoms in women undergoing prolapse surgery. Climacteric..

[84] Salinas-Casado J, Mendez-Rubio S, Esteban-Fuertes M, Gomez-Rodriguez A, Virseda-Chamorro M, Lujan-Galan M (2018). Efficacy and safety of D-mannose (2 g), 24h prolonged release, associated with Proanthocyanidin (PAC), versus isolate PAC, in the management of a series of women with recurrent urinary infections. Arch Esp Urol.

[85] Salinas-Casado J, Mendez-Rubio S, Esteban-Fuertes M, Gomez-Rodriguez A, Virseda-Chamorro M, Lujan-Galan M (2020). Large study (283 women) on the effectiveness of Manosar(R): 2 g of d-mannose + 140 mg of proanthocyanidins (PAC), of prolonged release. Arch Esp Urol.

[86] Sharma V, Nayak J, DeRossi C, Charbono A, Ichikawa M, Ng BG (2014). Mannose supplements induce embryonic lethality and blindness in phosphomannose isomerase hypomorphic mice. FASEB J.

[87] Lenger SM, Bradley MS, Thomas DA, Bertolet MH, Lowder JL, Sutcliffe S (2020). D-mannose vs other agents for recurrent urinary tract infection prevention in adult women: a systematic review and meta-analysis. Am J Obstet Gynecol.

[88] Kodner CM, Thomas Gupton EK (2010). Recurrent urinary tract infections in women: diagnosis and management. Am Fam Physician.

[89] De Nunzio C, Bartoletti R, Tubaro A, Simonato A, Ficarra V (2021). Role of D-mannose in the prevention of recurrent Uncomplicated cystitis: state of the art and future perspectives. Antibiotics (Basel).

